# Editorial: Current omics-based approaches as tools for improving the understanding, diagnosis and management of inflammatory lung disease

**DOI:** 10.3389/fmed.2023.1280282

**Published:** 2023-09-25

**Authors:** Paula Tejera, María E. Laucho-Contreras, Elizabeth Córdoba-Lanús

**Affiliations:** ^1^Departamento de Bioquímica, Microbiología, Biología Celular y Genética, Área de Biología Celular, Facultad de Ciencias, Sección de Biología, Universidad de La Laguna, San Cristóbal de La Laguna, Spain; ^2^School of Public Health, Harvard University Boston, Boston, MA, United States; ^3^Fundación Neumológica Colombiana, Bogotá, Colombia; ^4^Instituto Universitario de Enfermedades Tropicales y Salud Pública de Canarias (IUETSPC), Universidad de La Laguna, San Cristóbal de La Laguna, Tenerife, Spain; ^5^Centro de Investigación Biomédica en Red de Enfermedades Infecciosas (CIBERINFEC), Instituto de Salud Carlos III, Madrid, Spain

**Keywords:** chronic obstructive pulmonary disease (COPD), asthma, asthma-COPD overlap (ACO), cystic fibrosis, COVID-19, microbiota, system biology, omics

Omics technologies and their integration across multiple omics layers have revolutionized biomedical research. The combination of omics data helps researchers bridge the gap between genotype and phenotype by providing a more holistic and precise characterization of diseases, which, in turn, can result in personalized medicine and the development of more effective treatments. Omics and multi-omics approaches have been adopted by researchers in the field of inflammatory lung disorders, to understand disease and to identify novel therapeutic targets and pathways for intervention.

In this Research Topic we want to focus on highlighting new advances in the application of “omics” and systems biology approaches to the prevention, diagnosis, risk assessment, sub-phenotyping, progression, and treatment of inflammatory lung diseases.

Asthma and chronic obstructive pulmonary disease (COPD) are diseases that cause chronic inflammation of the airway. Clinical characteristics of both diseases can be simultaneously present in a given patient, a condition termed asthma-COPD overlap (ACO) (1). As of today, there are no molecular biomarkers that can assist clinicians in the differential diagnosis. In their study, Ma et al. explored hemogram-derived inflammatory indexes as biomarkers for distinguishing asthma, ACO, and COPD. The study reveals that patients with those conditions can be differentiated based on indexes of platelet, neutrophil-lymphocyte ratio (NLR), and eosinophil-basophil ratio (EBR). Besides hemoglobin and lymphocyte-monocyte ratio (LMR) were correlated with the severity of patients' symptoms, while platelet-lymphocyte ratio (PLR) and EBR were related to pulmonary function; nevertheless, the relationship between inflammatory indexes and patient outcomes could not be evaluated. The lack of a universally recognized specific definition of ACO and the small sample size of this study lead to cautious interpretation of the conclusions. Despite these limitations, the use of these hemogram-derived markers has several advantages, being cheap, easy to perform, and available in all health facilities. Further investigations will be needed to determine the clinical application value of these inflammatory indexes.

Combining multi-omics approaches at different scales has shown to be very useful in the characterization of inflammatory diseases status. COPD is a heterogeneous inflammatory disease, characterized by persistent respiratory symptoms and airflow limitation (2). In their study, Gao et al. explore the role of protein acetylation modification in relation to COPD using an *in vivo* mice model. Transcriptomics, proteomics, and acetylomics data of lung tissue were analyzed by RNA sequencing and liquid chromatography-tandem mass spectrometry. The results showed 19 genes to be differentially expressed at all the studied levels, simultaneously. From these, nine genes were involved in pathways related to mitochondria function. Subsequently, by single-cell RNA-seq they determined the distribution of the cited 19 genes in human lung tissue. Only *ALDOA* and *CORO1A* were differentially expressed in COPD patients with *ALDOA* widely expressed in various cell subpopulations which was also confirmed in mice lung tissue. The authors suggest that the downregulation and hyperacetylation of *ALDOA* might be a regulatory mechanism for mitochondrial damage and inflammation in response to cigarette smoke.

Systems biology is a holistic approach to deciphering the complexity of biological systems by analyzing the interactions and behavior of their components. To help uncover regulatory mechanisms, differential co-expression analysis studies diseases and phenotypic variations by finding modules of genes whose co-expression patterns vary across conditions (3). In their study, Queen et al., describe a novel method termed **a**ssociation of **c**ovariance for detecting **d**ifferential **c**o-expression (ACDC), able to detect differential co-expression across multiple binary, ordinal, or continuous phenotypes or exposures. The method is then applied to two independent cohorts of asthmatic patients to identify associations between gene co-expression and levels of asthma control. The results show associations between differential co-expression of inflammatory genes previously implicated in asthma and asthma control test scores. In comparison with the analysis using coXpress, a module-based differential co-expression method, the ACDC method allows for detecting differences in co-expression across levels of asthma control test scores. The ACDC method can also be applied to other types of molecular data, such as proteomics or metabolomics.

Among novel therapeutic approaches, the study of microbiota has emerged as relevant for the phenotypic characterization and the monitoring of disease therapies. Microbiota dysbiosis can lead to the dysregulation of many body functions and so, several diseases. Cystic fibrosis (CF) is a genetic disease where impaired pulmonary mucociliary clearance is produced with subsequent chronic airway infections and inflammation (4). In their study, Shumyatsky et al. performed a stratified analysis using metagenomic sequencing to determine the role that specific airway microbiome may play within each clinical state of CF patients at three-time points: pulmonary exacerbations (PEx), end of antibiotic treatment, and follow-up. Ten bacterial species with a differential gene abundance were found among which *Staphylococcus aureus* and *Streptococcus salivarius* were the most abundant in PEx and follow-up, respectively. These same species contributed mostly to the differential abundance of pathways. *Veillonella atypica* was increased in follow-up compared to PEx. Although the limited sample size the research findings highlight that the metabolic potential of bacterial species may explain more closely the changes across clinical states than the bacterial relative abundance alone. Longitudinal studies are needed to complement these findings.

In the same editorial line, in this issue, Li et al. present the results of an investigation of the relationship between SARS-CoV-2 infection and asthma using transcriptomics. Although, severe asthma has been associated with severe COVID-19 outcomes the relation between both diseases is still unclear (5). The researchers identified 66 differentially expressed genes between the two diseases. A protein-protein interaction (PPI) network that corresponded to five hub genes was constructed using several bioinformatics methods, and the online analysis website Search Tool for the Retrieval of Interacting Genes/Proteins (STRING). The core genes as *CLK4, CLK1, CHD1, CCNL1*, and *CCNT2* were identified as potential biomarkers that could be related to therapeutic targets in future research. The gene-disease analysis also performed showed a relationship of these genes with the dysregulation of the immune response. These findings open the vision toward the interrelationships between the given susceptibility that an individual may have against an acute infection and a genetic background that allows the development of severe disease. However, this study had some limitations associated with the lack of mechanistic and clinical research that are needed to confirm the theories above mentioned.

Finally, with this number the researcher can broaden his vision on the complex interactions between the endotypes of highly prevalent diseases such as asthma and COPD; rare diseases such as cystic fibrosis, and acute diseases such as COVID-19 caused by SARS-CoV-2. “Omics” approaches allow the evaluation of new strategies in diagnosis, treatment, prognosis, and prevention, which could well be carried out on a larger scale and more efficiently and completely compared to the performance of thousands of individual tests that are carried out for the identification of clinical and genetic targets. A few years ago, we could say that this was the future, but the unequivocal conclusion of this editorial is that this is the present that we must aim for in order to reach individualized medicine.

## Author contributions

All authors listed have made a substantial, direct, and intellectual contribution to the work and approved it for publication.
